# Enhancement of 3D-Printable Materials by Dual-Curing Procedures

**DOI:** 10.3390/ma14010107

**Published:** 2020-12-29

**Authors:** Xavier Fernández-Francos, Osman Konuray, Xavier Ramis, Àngels Serra, Silvia De la Flor

**Affiliations:** 1 Thermodynamics Laboratory, ETSEIB, Universitat Politècnica de Catalunya, Av. Diagonal 647, 08028 Barcelona, Spain; ali.osman.konuray@upc.edu (O.K.); xavier.ramis@upc.edu (X.R.); 2 Department of Analytical and Organic Chemistry, Universitat Rovira i Virgili, C/Marcel·lí Domingo s/n, 43007 Tarragona, Spain; angels.serra@urv.cat; 3 Department of Mechanical Engineering, Universitat Rovira i Virgili, C/Av. Països Catalans, 26, 43007 Tarragona, Spain; silvia.delaflor@urv.cat

**Keywords:** additive manufacturing, 3D-printing, dual-curing

## Abstract

Dual-curing thermosetting systems are recently being developed as an alternative to conventional curing systems due to their processing flexibility and the possibility of enhancing the properties of cured parts in single- or multi-stage processing scenarios. Most dual-curing systems currently employed in three-dimensional (3D) printing technologies are aimed at improving the quality and properties of the printed parts. However, further benefit can be obtained from control in the curing sequence, making it possible to obtain partially reacted 3D-printed parts with tailored structure and properties, and to complete the reaction by activation of a second polymerization reaction in a subsequent processing stage. This paves the way for a range of novel applications based on the controlled reactivity and functionality of this intermediate material and the final consolidation of the 3D-printed part after this second processing stage. In this review, different strategies and the latest developments based on the concept of dual-curing are analyzed, with a focus on the enhanced functionality and emerging applications of the processed materials.

## 1. Introduction

Additive manufacturing (AM) methods are gaining increasing popularity in recent years due to the development and growing availability of new processing equipment with decreasing cost and increasing production rates [1,2,3,4]. The possibility of building complex shapes and structures with a fine geometrical resolution makes it possible to produce designs with reduced use and waste of materials [4]. Customized parts of polymers, metals or ceramics can be produced without the need for molding or machining equipment [1]. These technological advances are also accompanied by the design and development of materials that meet the functional requirements of processed parts using other technologies in industrial environments [1,2,3,4]. However, these technologies are still focused on production of custom-made parts and small series rather than mass-production objects due to cost and productivity limitations in comparison with other conventional, well-established technologies [1,4]. In additional to productivity and economic considerations, AM techniques face additional challenges, in particular regarding material properties such as mechanical anisotropy, porosity, geometrical accuracy and appearance [4].

**Figure 1:**
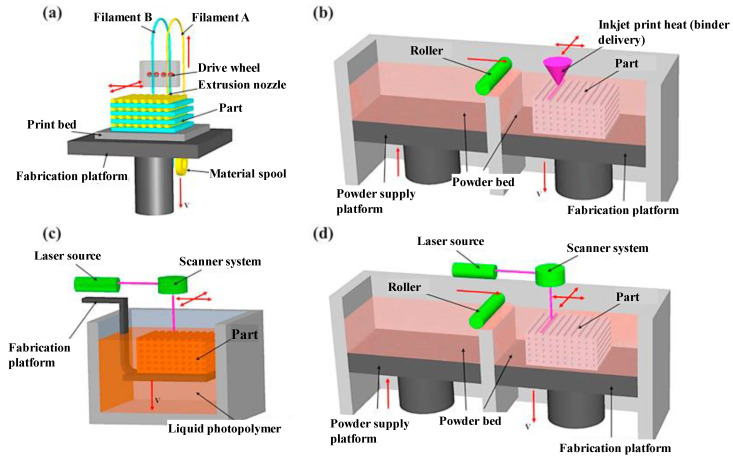
Schematic representation of some of the main additive manufacturing (AM) methods: (**a**) fused deposition modelling; (**b**) inkjet printing; (**c**) stereolithography; (**d**) powder bed fusion. Adapted from [6] with permission from Elsevier.

**Figure 2:**
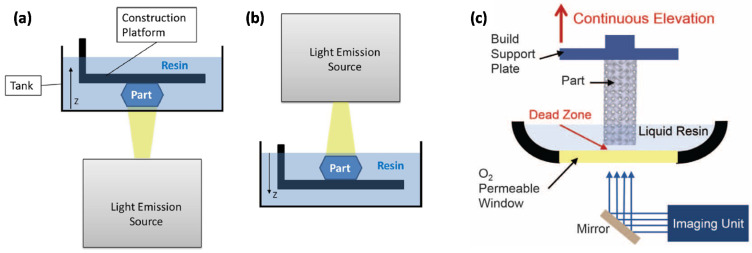
Schematics of digital light processing (DLP) following bottom-up (**a**) and top-down (**b**) approaches, and (**c**) continuous liquid interface polymerization (CLIP). (**a**,**b**) have been reprinted from [7] with permission from Elsevier, and (**c**) has been reprinted from [5] with permission from the American Association for the Advancement of Science.

A wide array of polymeric materials has been developed as new applications are emerging as a consequence of this technological development [1]. Thermoplastic materials are commonly used in popular fused deposition modeling (FDM) processes and also in powder bed fusion. Thermosets are usually processed by well-known 3D-printing technologies such as inkjet printing, stereolithography (SL) or digital light processing (DLP). SL and DLP are based on the layer-by-layer solidification of liquid or liquidlike thermosetting formulations that react upon exposure to ultraviolet (UV) or visible light leading to a crosslinked material. These techniques are advantageous in comparison with FDM because of the better resolution of the 3D-printed parts and enhanced mechanical properties [4]. Direct ink writing (DIW) is another technology used for the 3D-printing of thermosets, based on the combination of filament extrusion with monomer crosslinking [2] but, like FDM, it lacks the fine resolution of SL or DLP. Techniques such as continuous liquid interface polymerization (CLIP), based on DLP, represent a significant advance in terms of production speed overcoming one of the most severe limitations of these methods when a fine geometrical accuracy is required [5]. Schematics of all these abovementioned AM techniques are represented in Figure 1 and Figure 2.

Although DLP and SL are credited with producing components with high accuracy and mechanical properties, printed components still suffer from several drawbacks that limit their application. Nonuniform degree of cure throughout the printing direction leads to uneven and weak mechanical properties. High geometrical accuracy of the printed parts is often achieved at the expense of the completion of the curing process [7]. It is common practice to carry out a UV postcuring of the dry part after the 3D-printing stage in order to overcome these effects, but it is also acknowledged that UV light might be prevented from reaching the deepest layers of the material, therefore leading to regions with incomplete cure and nonuniform properties [8]. Excessive degree of postcure has been claimed to produce a negative effect as well [9]. Higher layer thickness can enhance productivity but, at the same time, have a negative impact on mechanical properties [10]. Mechanical properties and surface finish, among other quality parameters, are also influenced by part orientation, postcuring time/conditions and technology, and 3D-printing temperature [11,12,13,14].

Shrinkage, distortion and weak mechanical properties are important shortcomings of 3D-printed parts [9]. Curing shrinkage-induced stresses produced upon building and postcuring is responsible for creep distortions causing cracks and delamination [15]. This is a serious drawback because acrylates and methacrylates are mainly used in 3D-printing formulations for processing speed, but they are also known to have significantly higher curing shrinkage than other thermosetting systems, such as those based on ring-opening polymerization [1,9]. Larger monomers or oligomers with lower curing shrinkage can be employed [1], but this requires the use of a heated printing stage to reduce the viscosity to suitable levels for the printing process [13].

Productivity limitations can be overcome by fast processing techniques such as CLIP, which relies on oxygen inhibition during radical polymerization of acrylates/methacrylates for the control of the layer build-up [5]. However, this prevents the use of other UV-curing systems such as those based on the cationic ring-opening of epoxides. In addition, radical systems react in general much faster than epoxides [16,17,18], which makes them also more attractive in terms of productivity. Extinction of radical species is fast due to termination reactions, which makes it possible to control the penetration of light and extent of cure and, in consequence, layer thickness and printing accuracy during the process by selecting adequate light intensity/exposure time parameters. In contrast, cationic UV-curable systems exhibit significant dark cure [16], which makes them also attractive as components in 3D-printing applications. The combination of radical and cationic systems into 3D-printing formulations is aimed at benefiting from the advantages of both systems [9].

The combination of different polymerization reactions in thermosetting systems has attracted interest in past years. This is found in the concept of interpenetrating polymer networks (IPNs) [19], the combination of two or more polymers in which at least one of them is crosslinked. IPNs have been largely investigated in recent years [16,19,20,21,22,23,24,25,26,27]. The formation of different polymer networks can be a complex phenomenon involving a phase separation and different gelation events, leading to different structural and morphological characteristics and properties depending on polymerization sequence, composition and polymer compatibility, among other factors. Because of that, IPNs are claimed to lead to interesting and/or superior properties in comparison with the individual polymer components.

**Figure 3:**
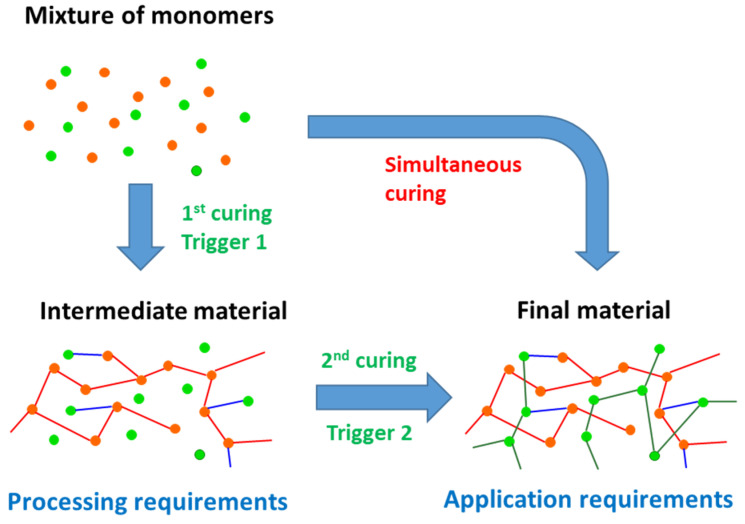
General representation of dual-curing processing.

The dual-curing concept [28,29] involves the combination of two polymerization processes in a single thermosetting formulation, which can be activated simultaneously or, preferably, in a sequential and controlled way, using selective triggers such as UV light or temperature or by differences between the curing kinetics of the two processes (Figure 3). It should be noted that dual-curing is somehow evolved from that of IPNs, in that two polymerization processes are combined in a single thermosetting system. However, an IPN structure is not necessarily obtained in a dual-curing process; in fact, final materials with an IPN or hybrid network structure can be obtained depending on the choice of reactions and monomers and the possibility of extensive covalent bonding between the two polymer networks. In addition, it is not only the final morphology, structure and properties resulting from such a combination that dual-curing focuses on, but also the control of the curing sequence and the processing possibilities arising from the existence of an intermediate material with controlled structure and properties. Unlike other multistage systems such as B-stage formulations, which are subject to tight time/temperature restrictions in order to control the properties reached after the predrying or precuring step before the final application, sequential dual-curing systems enable the formation of stable and well-defined intermediate materials after completion of the first curing stage [28]. The intermediate extent of reaction and the network/molecular architecture and properties of the intermediate and final materials can be easily tuned by means of changing the monomer feed ratio and architecture [30]. Overall, these features make sequential dual-curing systems highly attractive from a processing point of view. Dual-curing systems were developed in order to cure at elevated temperatures unexposed areas in photopolymerization processes [31]. Sequential systems can be used as dry-bond adhesive in microfluidics applications [32] or to produce autonomous temperature-triggered actuators [33,34]. Reshaping ability of the intermediate materials can also be used for the assembly of complex shapes or the production of complex shape structures [35]. Control of the intermediate extent of curing can be exploited in some composite processing applications [36]. Advanced optical applications based on dual-curing systems have also been reported [37]. Hybrid organic-inorganic materials obtained by a combination of curing and sol-gel process can also be categorized as dual-curing systems [38,39,40,41].

Another interesting application of dual-curing systems can be found in the area of 3D-printing. So far, the applications and possible developments have scarcely been mentioned in recent reviews [1,9]. Hybrid dual-cure formulations combining radical homopolymerization of acrylates and cationic polymerization of epoxides are recognized to enhance significantly the quality of printed parts due to the reduction in shape distortion and to the further postcure after building [1] and are indeed routinely used in 3D-printing applications. However, the scope of dual-curing systems in this area is much broader, especially for sequential systems, taking into consideration the wide range of possible combinations of polymerization reactions, the control of the material structure and extent of reaction after 3D-printing, and the change in structure and properties after the activation of the second polymerization or post-polymerization processes. Different dual-curing strategies, along with their benefits and applications, are considered in this review: (a) re-activation of the radical polymerization by means of a thermal radical initiator, (b) simultaneous activation of different polymerization processes in the 3D-printing stage, (c) a combination of different polymerization processes with sequential and controlled activation in the 3D-printing and a post-processing stage, and (d) other post-polymerization processes. The latest developments in this area are analyzed, focusing on the benefits obtained in terms of material properties, quality, and enhanced applicability and functionality of the 3D-printed parts.

## 2. Sequential 3D Printing and Thermal Radical Polymerization

The first dual-curing approach that we consider consists of the combination of UV-curing and thermal curing using radical photoinitiators and thermal radical initiators for acrylate/methacrylate homopolymerization. Strictly speaking, both initiators activate the same polymerization reaction, so it does not fit exactly the definition given in the introduction of a dual-curing system. However, the reaction must be activated twice using different triggers, UV light and temperature, and requires the use of different initiators for each trigger. In the absence of the supplementary thermal radical initiator, a thermal treatment alone would not be able to resume and complete the polymerization process because of the fast extinction of radicals, unlike cationic systems.

**Figure 4:**
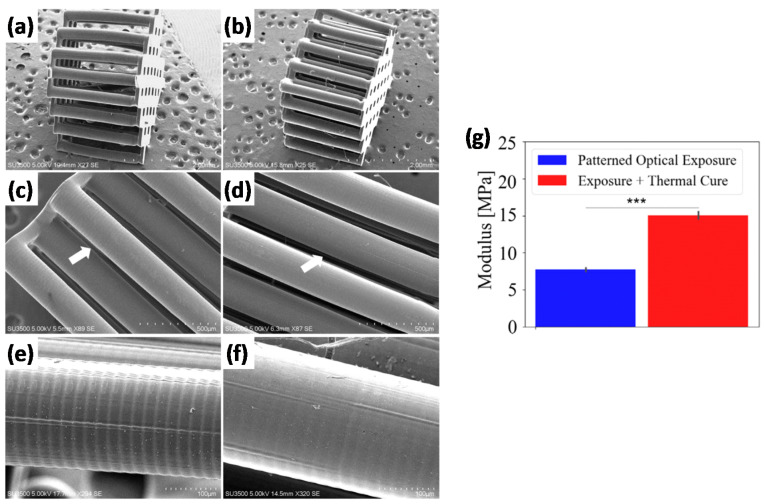
(**a**–**f**) SEM images for multilayer 3D printed parts fabricated via SL: (**a**,**c**,**e**) photo-cured structure compared to (**b**,**d**,**f**) thermally postcured multilayer samples; the arrows in (**c**,**d**) identify the pillar structure shown in (**e**,**f**). (**g**) Effect of thermal treatment on the mechanical properties of the multipillar structure. Extracted from [8] with permission from Wiley.

The use of an additional radical initiator was first reported by Gupta et al. [42], with the purpose of achieving complete and uniform cure in carbon fibers reinforced composites processed by rapid prototyping. This was required as carbon fibers employed in the SL resin prevented full cure due to the opacity of these fibers to UV radiation. A peroxide initiator was added to an acrylate/methacrylate-based SL formulation, which would not be activated during the SL process. High tensile strength specimens were obtained after the SL process due to the presence of the carbon fibers, but strength further increased after thermal postcuring up to 30% due to the reaction of residual unreacted monomer. The matrix-fiber adhesion was greatly enhanced due to the curing of unreacted liquid trapped inside the carbon fiber tow after the SL process. The thermal postcuring process was carried out at a temperature lower than the ultimate glass transition temperature of the material so that the materials obtained would be vitrified after the process, but complete cure might not have been reached. Materials with tack-free surfaces were obtained in any case after the treatment. Following a similar approach, Uzcategui et al. [8] modeled the photopolymerization process of acrylate formulations modified with thiol groups and the effect of thermal postcuring by means of an added thermal initiator. The authors studied in detail the expected properties across the thickness of printed parts and showed that the thermal postcuring produced materials with bulk properties comparable to those of the more irradiated (and more cured) layers. In addition, thermal treatment produced smoother surfaces by reduction of the ridging on the surface of printed samples caused by the differential shrinkage/swelling produced after printing. Both the enhancement in mechanical properties and the smoothing effect produced by the thermal treatment are illustrated in Figure 4. However, it was also noted that, because traces of unreacted monomer might be still present in the sample, maximum properties could only be obtained if unreacted monomer was eventually removed from the material by soaking the sample in a suitable solvent after the 3D printing and a thermal treatment. Konuray et al. [43] also reported that ultimate material properties could only be obtained if a thermal radical initiator was added in order to complete the reaction of acrylate monomers in dual-curing formulations for stereolithography.

**Figure 5:**
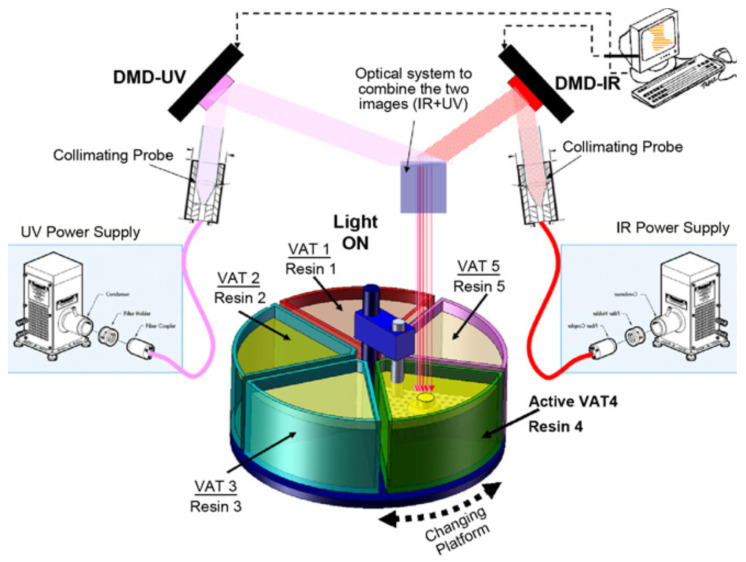
The micro stereo-thermal-lithographic process: multivat system. Reproduced from [46] with permission from Elsevier.

Following a somewhat different approach, Bartolo and Mitchell [44] outlined a method for simultaneous UV-thermal curing using a combination of photo and thermal radical photoinitiators (stereo-thermal lithography, STLG). The authors claimed that this process was more effective because the whole process could be carried out in a single stage, rather than a printing followed by postcuring stage, and in addition it could prevent further distortion produced in the postcuring stage. The proportion of the radical photo and thermal initiator was considerably lower than in conventional UV-curable and thermally curable formulations, so that the individual effects of light or temperature would not activate the curing process; however, at the selected light-intensity and temperature conditions acting simultaneously, the generation of radicals would be sufficient so that the polymerization would be carried out. The authors first outlined the method and tested it using a simple photomask and heated plate system [44], but it is being developed into an operating micro stereo-thermal lithography (µSTLG) device combining UV and infrared (IR) radiation [45,46] with the possibility of producing multimaterial structures as it is schematically represented in Figure 5.

All these approaches, based on the completion of radical homopolymerization of acrylates by means of a second thermally activated process, help to overcome one of the most relevant drawbacks of acrylate-based 3D-printing processes, that is, the lack of uniformity of the printed materials arising from the layer-by-layer build-up process and the incompletion of the curing process. In consequence, uniform and isotropic materials, with enhanced and predictable properties, are obtained. Although UV postcuring processes are customarily used to overcome this drawback, this additional thermal treatment with the help of a thermal initiator may be necessary because of the poor penetration of UV light in the deepest layers of material, especially in the presence of fillers that may block radiation.

## 3. Simultaneous Dual-Curing Systems Combining Two Polymerization Mechanisms

The simultaneous activation of two polymerization reactions by exposure to UV light during the printing stage is the best-known application of the dual-curing concept in 3D-printing. Typically, acrylate/methacrylate radical homopolymerization is combined with cationic homopolymerization of epoxides, in producing an IPN structure. Radical and cationic photoinitiators must be included in the formulation. 3D-printed material hybrid materials exhibit significant dark cure in a subsequent thermal treatment, because of the presence of unreacted epoxy groups and trapped cationic active species in the material, which enable subsequent reaction after heating and devitrification of the printed material. This extended reaction can lead to more uniform and isotropic materials, hence improving the quality of printed parts [47]. Hybrid radical-cationic 3D-printed materials present reduced curing shrinkage and warping effects [9,48]. Residual stresses in epoxy-based SL printed parts are much lower than that of acrylate-based materials as investigated by Karalekas et al. [15,49]. Thermal treatments to complete the reaction of unreacted epoxy monomers increase the level of residual stress, but it still remains considerably below that of acrylates.

Fillers can be incorporated into hybrid radical-cationic 3D-printing formulations in order to enhance their performance. Esposito Corcione et al. [50] tried to improve the properties of dual-curable acrylate-epoxy formulations for SL by means of the addition of montmorillonite fillers. SL-printed samples were not completely cured due to vitrification. Thermal treatment of the samples did enhance the thermal properties of the materials due to the dark reaction of epoxides, but the effect of the filler was unclear. In contrast, Kumar et al. [51] found a good compatibility between the thermosetting polymer matrix and cellulose nanocrystals caused by reaction of hydroxyl groups with the epoxy monomers, leading to a significant enhancement in the mechanical strength of the materials. In that case, a UV post-treatment was used instead of a thermal postcuring.

Invernizzi et al. [52] developed silica nanocomposites with low shrinkage starting from hybrid formulations for stereolithography combining epoxy and acrylate monomers. The photoinitiation system consisted of a cationic photoinitiator capable of producing also radical, and a photosensitizer to enable activation upon laser epoxsure in the near-visible range. The authors used 0.5 wt.% of zeolites as a moisture scavenger and to prevent deactivation of the cationic photoinitiator. The authors reported the formation of a phase-separated IPN after printing, but thermal treatment produced materials with apparently a single-phase structure due to a temperature-induced solubilization of the networks or the formation of a metastable phase. Printed samples exhibited very little shrinkage after one-week storage in comparison with commercial SL formulations. Similarly, Cui et al. [53] employed urethane epoxidized soybean oil in 3D-printable formulations with acrylates. The effect of the epoxy comonomer on the microstructure of the material was not discussed, but the authors reported that the use of the epoxide comonomer reduced the total curing shrinkage of the cured materials, and the thermal-mechanical properties were enhanced in comparison with the unmodified material.

**Figure 6:**
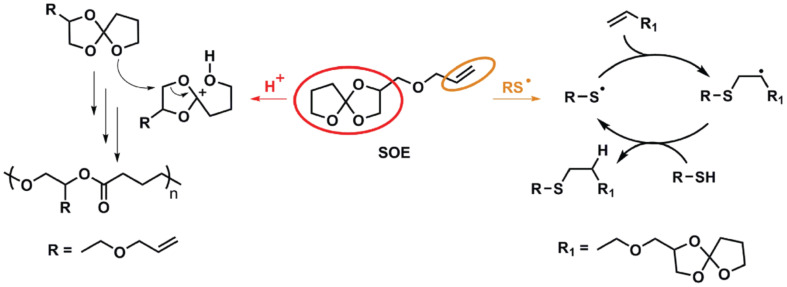
Mechanism of the cationic double ring-opening and the thiol-ene reaction of the spiroorthoester (SOE). Reproduced from [55] with permission from Wiley.

Polymerization of bicyclic spiranic structures based on spiroorthoester (SOE) or other monomers is known to proceed with very little or no shrinkage at all [54]. Following this distinct approach, Marx et al. [55] combined radical thiol-ene with cationic ring-opening of an SOE to produce low-shrinkage materials with good material permittivity. An SOE-modified ene monomer was synthesized and incorporated into a thiol-ene formulation (see scheme in Figure 6), leading to a relevant decrease in curing shrinkage by the cationic tandem ring-opening of the SOE. In addition, the printing resolution was improved with the addition of this SOE comonomer.

**Figure 7:**
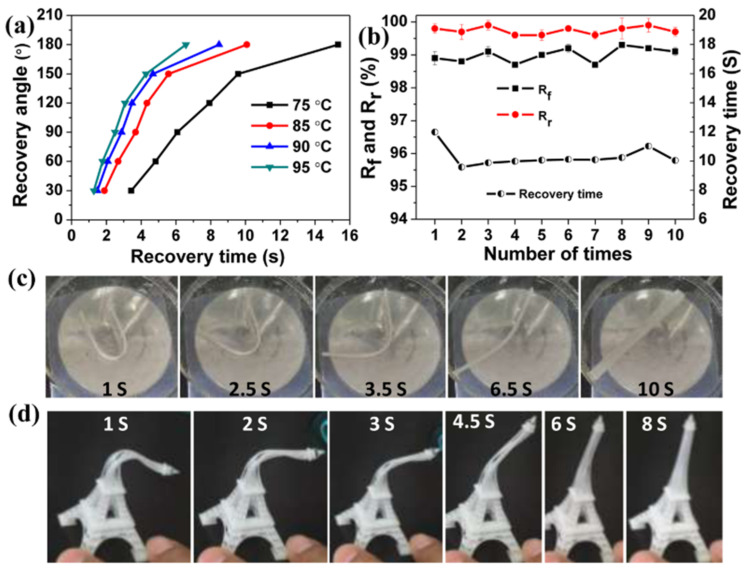
(**a**–**c**) Results (recovery angle, fixity ratio R_f_ and recovery ratio R_r_) and visual representation of the of shape-memory fold-deploy tests for printed hybrid acrylate-epoxy samples, and (**d**) visual demonstration of the shape-recovery process of a complex printed shape. Reproduced from [56] with permission from ACS Publications.

3D-printable hybrid materials have also been studied with the purpose of producing shape-memory materials. The approach followed by Yu et al. [56] consisted of the preparation of preformed acrylate-epoxy particles that were incorporated into a hybrid formulation containing acrylates, and epoxy and oxetane monomers. Radical and cationic photoinitiators were used in the formulation. A co-continuous IPN structure after printing was obtained, although phase separation was observed as a consequence of the presence of the preformed particles. Thermal postcuring, producing dark cationic curing of unreacted epoxy/oxetane groups, led to materials with properties apparently more uniform than the printed ones. The presence of preformed particles did also impart significant toughness to the resulting materials. Shape-memory properties were remarkable, showing quantitative fixity and recovery ratios (Figure 7), and were comparable to other materials processed by conventional means reported in the literature.

While the combination and simultaneous activation of two polymerization reactions can contribute greatly to improve the properties of printed parts, such hybrid systems exhibit some limitations: (a) the activation of the cationic polymerization precludes the use in CLIP-based fast printing stages, and (b) the range of polymerization reactions that can be combined is very limited. Such limitations can be overcome in sequential dual-curing systems, and additional benefits can be derived from the processing and application point of view.

## 4. Sequential Dual-Curing Systems Combining Two Polymerization Mechanisms

Sequential dual-curing systems for 3D-printing formulations differ from the previous examples in that only one of the polymerization reactions is activated during the 3D-printing stage, while the second polymerization process is activated generally by means of temperature and a suitable initiator (if needed). In consequence, the possibilities of a combination of different curing processes are significantly widened since they are not limited to the availability of photoinitiators. This also broadens the range of properties that can be achieved in 3D-printing processes based on dual-curing. It will be seen that most dual-curing systems reported make use of an epoxy reaction for the second polymerization process, but other systems have been successfully demonstrated.

Sequential dual-curing systems for 3D-printing have been recently developed by Carbon, Inc. [57] and are commercially available for use in their proprietary printers based on the continuous liquid interface production (CLIP) system [5,58], a concept termed as digital light synthesis (DLS). However, few reports on their use and properties are available in the literature.

Obst et al. [59] analyzed the effect of light exposure on the dual-curing processing of commercial formulation RPU 70 from Carbon, Inc. (Redwood City, CA, USA) and the resulting material properties. This material is a two-component system, A and B, which is only mixed prior to processing to prevent dark reaction upon storage. The A component contains a photo-crosslinkable diurethane dimethacrylate oligomer, while the B contains a triamine component that will participate in the network formation of the second reaction at elevated temperature, producing the breakage of the diurethane structures and transformation into diurea structures constituting a new polyurea network. An additional dimethacrylate oligomer is incorporated into the B component to ensure that a methacrylate network remains after the second reaction takes place. The authors reported that longer irradiation times for the radical homopolymerization of methacrylates apparently reduced the extent of the thermal crosslinking stage. The thermal treatment produced a significant increase in tensile strength and a certain increase in elongation at break, except for the samples that were exposed longer to irradiation, for which a significant decrease in elongation at break after the thermal treatment was observed. Values of elastic modulus were not reported. According to the technical data sheet of this product, RPU70 is claimed to have a heat deflection temperature of about 60 °C and a glass transition temperature (tan δ peak) of about 125 °C, but the authors did not show the effect of processing conditions on the glass transition temperature of the materials.

**Figure 8:**
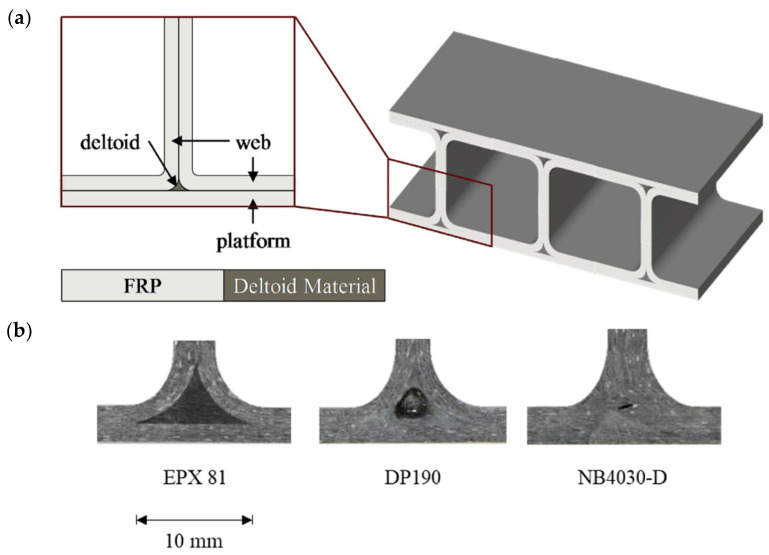
(**a**) Schematic cross-section of a T-joint (left) and schematic 3D drawing of a spar-wingskin joint (right). (**b**) Close-up of deltoid areas for T-joints filled with the EPX 81, a commercial epoxy adhesive (DP190) and a prepreg inserted. It can be seen how the deltoid manufactured with the 3D printed dual-cure epoxy shows an exact geometry and a good material distribution along the bond line without voids or discontinuities. Reproduced from [61] with permission from Elsevier.

Dual-curing formulation EPX82 from Carbon, Inc. (Redwood City, CA, USA) was studied by Redmann et al. [60]. This formulation is based on acrylate homopolymerization for the first 3D-printing stage and, according to the technical data sheet of EPX82, the second reaction is an aromatic epoxy/amine reaction, but details are not disclosed. Intermediate printed materials were soft gels with a glass transition temperature of 14 °C, which increased up to 155–160 °C after the epoxy reaction was carried out in the thermal treatment, which consisted of a slow multistep curing process at temperatures up to 220 °C. The authors optimized the thermal curing process from 9 h to 3 h without compromising the thermal-mechanical properties, while controlling the maximum conversion rate to moderate values. Later on, Dahmen et al. [61] studied the application of a similar EPX 81 system exploiting the adhesive properties of the intermediate, partially cured material for the fabrication of hybrid composite T-joints. Specimens for lap-shear tests were prepared using printed adhesive layers that were sandwiched between prepreg plies on each side and cured in an oven under vacuum to ensure good contact within the prepreg plies, and between the prepreg plies and the adhesive layer. The maximum shear stress was comparable to other commercial adhesive systems. Samples prepared for testing of T-joint adhesion with the EPX81 material showed much higher geometrical accuracy than other adhesive systems due to the geometrical and mechanical stability of the printed adhesive part (see Figure 8), and the pull-out strength was comparable to the other commercial adhesives. The intermediate adhesive properties were also exploited by Chen et al. [62] with the purpose of producing printed bike saddle structures using EPX82 material and reinforced in the most vulnerable areas by co-curing with preimpregnated carbon fiber under vacuum.

**Figure 9:**
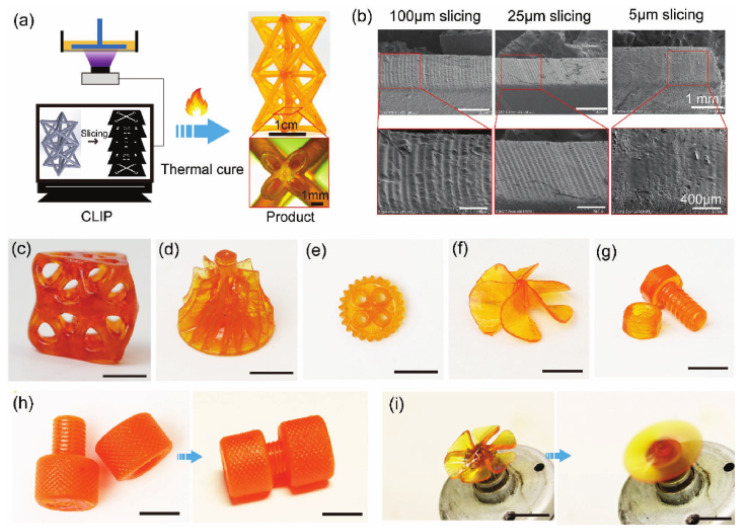
CLIP-assisted DLP 3D printing of interpenetrating polymer network (IPN) epoxy composites via two-stage curing and the potential applications as structural materials. (**a**) CLIP assisted fabrication of thermosets. (**b**) SEM images of patterns by CLIP-assisted fabrication at the same print speed and different slicing layer thickness. (**c**–**i**) Diverse architectures produced, in particular (**h**) a nut-screw set before and after assembling and (**i**) a printed fan blade driven by a motor. The scale bar is 1 cm in (**c**–**i**). Reproduced from [63] with the permission of Wiley.

Kuang et al. [63] used a homemade 3D-printer based on CLIP technology in order to study the high-speed 3D-printing of a noncommercial dual-curable formulation based on an acrylate and epoxy-anhydride components for the printing and thermal postcuring stages, respectively. Glycidyl methacrylate was used as reactive diluent and coupling agent, and an amine-based initiator was used as a catalyst for the activation of the epoxy-anhydride reaction upon a thermal treatment at 100 and 160 °C. Curing shrinkage was very high because the authors used low molecular-weight acrylate monomers to control the viscosity of the acrylate formulation, but it was reduced upon addition of epoxy comonomers, from 26% for the neat acrylate to 10% for the IPN containing 80% of the epoxy network. Final IPN materials had glass transition temperatures within the range of 140–160 °C with no macroscale phase separation, which could be explained by the presence of the coupling agent. The presence of the epoxy network resulted in materials with comparable modulus but considerably higher tensile strain and strength in comparison with the neat acrylate and neat epoxy components. The authors showed the application in electronics devices and structural materials, as shown in Figure 9.

**Figure 10:**
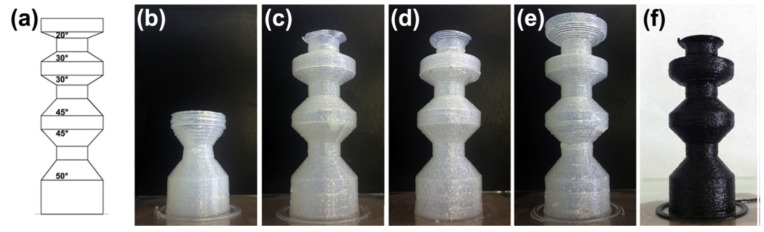
3D printed models with the inks obtained in [64]. (**a**) Layout of the target model (**b**–**e**) printed samples with increasing proportion of the photocurable acrylic resin and (**f**) adding carbon fibers. Reproduced from [64] with permission from Elsevier.

Griffini et al. [64] studied dual-curing systems based on photocurable acrylate and thermally curable epoxy-dicyandiamide (epoxy-DICY) catalyzed with diuron. Fumed silica was used to control the viscosity of the resins so that they could be processed by direct ink writing (DIW). Samples with a minimum of 5 wt.% of photocurable acrylate component could be printed, but at least 20 wt.% of the acrylate was required to print steep overhanging features (Figure 10). The presence of the epoxy component increased the glass transition temperature and elastic modulus of the printed materials after thermal post-processing at 220 °C. Reinforcement with carbon fiber (CF) was studied, requiring a presence of at least 50 wt.% of photocurable acrylate to make up for the lower photopolymerization efficiency caused by the blocking of UV light. IPNs with an apparently single mechanical relaxation were obtained, possibly explained by the good compatibility between the bisphenol A backbone of both the epoxy and acrylate monomers. Incorporation of CF also improved the mechanical properties, but the effect was lower than expected. Using a similar printing platform, Invernizzi et al. [65] studied the effect of epoxy-anhydride as a thermally curable second component of 3D-printable formulations reinforced with glass fiber (GF) or carbon fiber (CF). The use of the epoxy-anhydride enabled the lowering of the temperature for the thermal postcuring down to 140 °C. Materials with higher epoxy content had somewhat lower elastic modulus but higher tensile strength and elongation at break. Good matrix-reinforcement interaction enabled an effective enhancement of the elastic modulus and tensile strength using both CF and GF.

**Figure 11:**
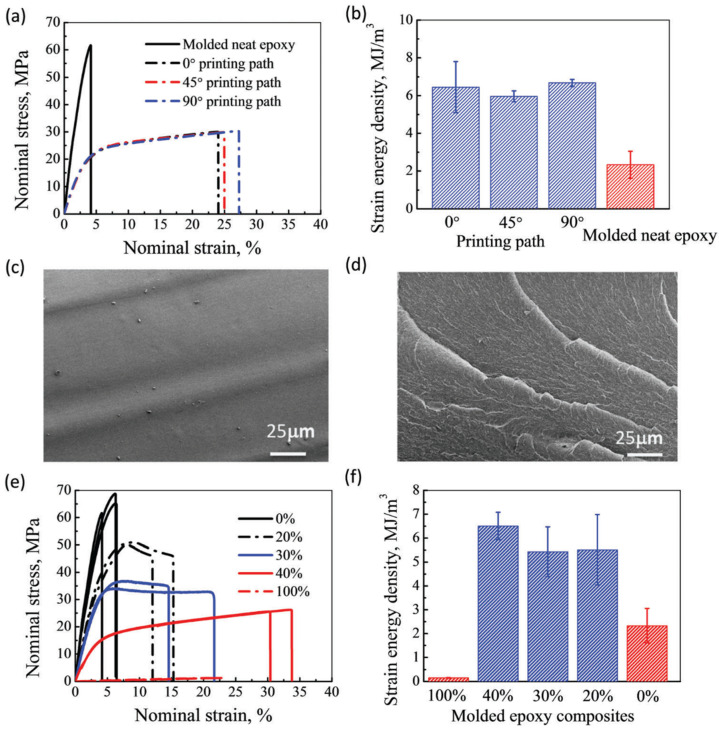
(**a**,**b**) The stress-strain curves and strain energy density of the molded neat epoxy and print epoxy composites with different printing paths, respectively; (**c**,**d**) the SEM of the fracture surface of the molded neat epoxy and print epoxy composite material with 0° printing path, respectively; (**e**,**f**) the stress-strain curves and the strain energy density of molded epoxy composites with different weight fractions of photopolymer, respectively. Percentage is for the photocurable resin. Reproduced from [66] with permission with permission from the Royal Society of Chemistry.

Chen et al. [66] also used DIW to produce acrylate-epoxy hybrid materials. An acrylate-based formulation with a radical photoinitiator for 3D-printing was modified with 60 wt.% of a bisphenol A-based epoxy monomer thermally curable with an amine-based anionic initiator. Nearly isotropic materials were obtained after the printing process and a subsequent UV and thermal treatment were completed, due to the merging of adjacent printed filaments and good interfacial adhesion between layers. Figure 11 shows that the hybrid materials were tougher than soft acrylate and hard epoxy components, as determined from the total strain energy density in stress-strain measurements, and had glass transition temperatures in the range of 60–90 °C. These mechanical properties made the materials suitable for shape-memory applications.

Konuray et al. [43] developed dual-curable formulations based on commercial acrylate resins for stereolithography modified with diepoxy monomer, with storage stability of up to 2 months at 32 °C, making it suitable for the preparation of stable one-pot formulations. Storage stability and control of the curing sequence were achieved by means of the use of (1) a radical photoinitiator for the 3D-printing stage and (2) a latent cationic initiator based on quaternary ammonium salts stabilized with a tertiary amine for the thermal cationic homopolymerization of the epoxy resin. A latent thermal radical initiator was also added in order to maximize the degree of cure of the acrylate fraction and therefore ensure final uniform material properties. Materials with IPN structure and good compatibility between the two polymeric networks were obtained after the 3D-printing and thermal postcuring process. An increase in the elastic modulus of the material was reported due to a systematic increase of the glass transition of the resulting IPNs with increasing epoxy content.

A different control of the curing sequence was attempted by Lantean et al. [67]. Radical and cationic photoinitiators were chosen so that selective wavelength irradiation could produce sequential curing, i.e., visible light for the acrylate homopolymerization during 3D-printing followed by UV postcuring for the cationic homopolymerization of epoxides. However, radical-induced activation of the cationic photoinitiator led also to cationic homopolymerization in the 3D-printing stage when the radical photoinitiator concentration was high. The presence of the epoxy network increased the brittleness of the materials and reduced the elastic modulus of the materials. The use of an epoxy-methacrylate coupling agent produced materials with high fracture toughness at optimum concentrations. The effect on mechanical properties and morphology was complex, but separate network relaxations corresponding to the acrylate and epoxy networks could be observed at higher epoxy concentrations.

**Figure 12:**
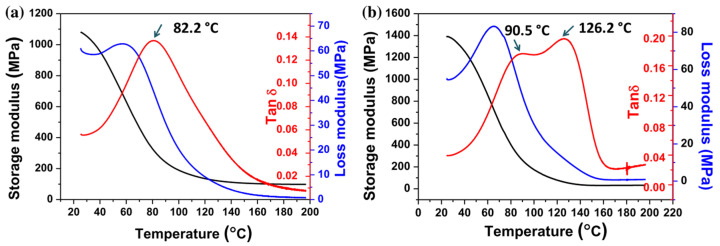
DMA results of the printed samples from (**a**) pure photopolymerization system and (**b**) photo-thermal dual-curing system. Reproduced from [68] with permission with permission from Springer.

Zhao et al. [68] compared the simultaneous UV-UV curing and sequential UV-thermal curing of acrylate-epoxy formulations for 3D-printing using an SL machine. The authors synthesized a silicon-based diepoxy monomer that they employed in combination with acrylate monomers in simultaneous dual-curing processes using radical and cationic photoinitiators. A UV postcuring and a thermal postcuring were applied on the samples to ensure complete conversion of acrylate and epoxy groups. They compared this system with sequential dual-curing systems using the same acrylate and epoxy monomers, but adding an anhydride as a curing agent for the epoxy monomer. They used a radical photoinitiator for the 3D-printing stage and an imidazole as an accelerator for the thermal epoxy-anhydride reaction. The inspection of SEM micrographs of the samples and the DMA characterization (see Figure 12) revealed that the simultaneous curing led to uniform materials with co-continuous nano-sized IPN structure, while sequential curing led to phase-separated opaque materials with micro-sized epoxy domains. The photo-thermal sequential materials had higher modulus and mechanical strength, but comparison between the different curing procedures are difficult because of the different epoxy curing mechanism and network structure produced.

**Figure 13:**
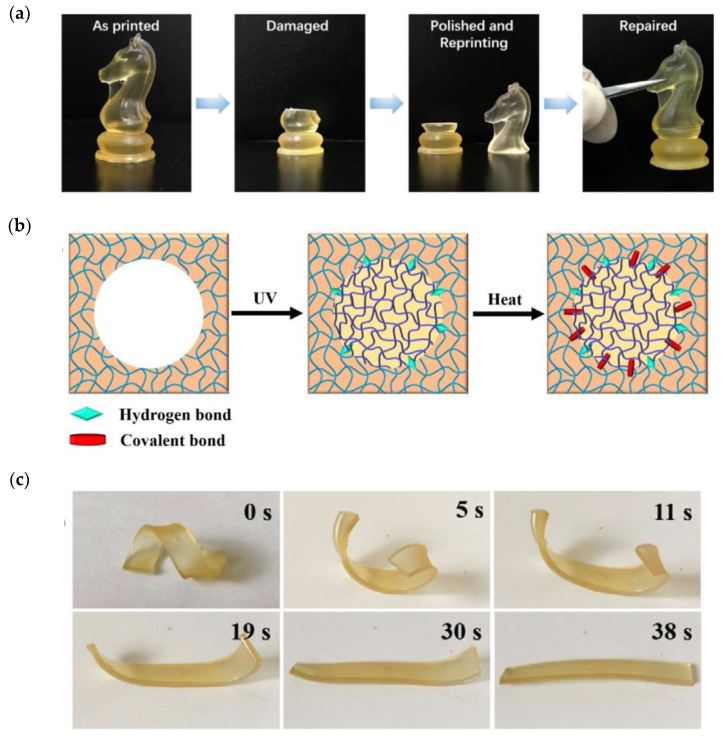
(**a**) Visual demonstration of the repairability and its mechanism (**b**,**c**) of the shape memory ability, of the 3D printed thermosets of Lu et al. [70]. Reproduced from [70] with permission from Wiley.

Following a completely different approach, Zhou et al. [69] described a dual-curing system based on an acrylic mixture containing OH-functional dimethacrylate produced by reaction of glycidyl methacrylate with adipic acid for 3D-printing stage using DLP, which was co-cured with PHDI (isocyanurate of hexamethylene diisocyanate) in a subsequent thermal curing process at 120 °C, leading to the formation of a polyurethane co-network by reaction with OH groups of the dimethacrylate monomer. The printing formulation was not latent, leading to a slow increase in viscosity upon storage at 25 °C. PHDI had a limited effect on thermal and mechanical properties, with a moderately low optimum concentration, because polyurethane formation was incomplete at higher PHDI content by topological restrictions after formation of the acrylate network in the 3D-printing stage, and reaction was incomplete. Lu et al. [70] used a similar reaction scheme to produce 3D-printable objects from renewable sources, with fluorescent and shape-memory properties. A methacrylate macromonomer derived from cellulose in combination with OH-containing monomers, a rosin methacrylate derivative and hydroxyethyl acrylate were used for the 3D-printing stage using a suitable radical photoinitiator. Hexamethylene diisocyanate (HDI) was used for the subsequent thermal reaction at 120 °C for up to 8 h, leading to the formation of a polyurethane co-network by reaction with the different hydroxyl groups available in the printed material. The amount of HDI employed was limited to a 5% molar ratio with respect to the hydroxyl group content of the material, to ensure complete isocyanate reaction. The second reaction increased the glass transition temperature of the printed material and produced a broadening of tan δ traces (DMA), which the authors attributed to phase separation. The 3D-printed materials had interesting repairability properties, producing almost identical materials after replacement of missing parts by freshly printed specimens and adhesion by thermal co-curing, made possible by the large amount of available hydroxyl groups in the material (Figure 13a,b). The materials also had remarkable shape-memory behavior (Figure 13c). The presence of rosin structure in the materials conferred fluorescent properties. Conductive hydrogels retaining the three-dimensional structure of the printed material could also be obtained by hydrolytic degradation.

The use of benzoxazines in 3D-printing dual-curing systems has been also recently reported in the literature. Weigand et al. [71] synthesized a bisphenol A-benzoxazine-dimethacrylate monomer and used it in combination with an acrylate reactive diluent for viscosity control. A thermal postcuring process was carried out at 200 °C to promote benzoxazine ring-opening. Final materials with broader relaxations at temperatures of about 100 °C were obtained, with hybrid polymer network or co-network structure.

In summary, sequential dual-curing systems exhibit much more variety than simultaneous hybrid dual-curing networks, because of the wider range of polymerization reactions that can be used for the second processing stage after the 3D-printing step, which is based on acrylate/methacrylate radical homopolymerization. However, most of the systems reported make use of epoxy reactions for the second curing stage because of the high versatility of epoxy groups in terms of reactivity. The properties of dual-curing systems, mostly IPNs from a structural point of view, are generally dependent on the contribution of the different polymer networks. Given the large combination of reactions, monomers, coupling agents and formulation proportions, it is almost impossible to derive any systematic analysis in comparison with other curing systems, but all the same it shows that the tailoring ability of sequential dual-curing systems is vast. Moreover, it has been seen that the control of the intermediate material structure can lead to interesting adhesion and healing abilities of the 3D-printed parts, which can be exploited for a number of applications. This is possibly one of the strongest advantages of these systems, in comparison with conventional or hybrid simultaneous dual-curing 3D-printable materials. The key for the success of their application is the control of the curing sequence and storage stability of these systems using suitable latent catalysts.

## 5. Other Multiple-Stage 3D-Printing Systems

So far, we have reviewed organic polymer-based dual-curing systems leading to IPN or hybrid network structures (organic), in which all the components were already present in the printing formulation. However, it is possible to incorporate inorganic network structures within the material by a second-stage processing, to incorporate externally other components with the purpose of completing the reaction and/or achieving specific properties, or else produce a rearrangement of the network structure of the material, therefore changing its properties after treatment. Production of 3D-printed ceramic components generally involves a pyrolytic treatment in order to remove the organic binder photocured in the 3D-printing process and to produce sintering of the ceramic particles [1,4], but these systems will not be discussed.

Hybrid organic-inorganic networks were also produced by Chiappone et al. [72]. The authors modified PEG-based acrylate formulations with inorganic precursors, 3-(triemethoxysily) propyl methacrylate and TEOS. Three-dimensional objects were printed using DLP and were subsequently subjected to thermal post-processing at 70 °C under acidic humid conditions in order to carry out a sol-gel reaction at 70 °C with the purpose of producing an in situ reinforcement of the material.

**Figure 14:**
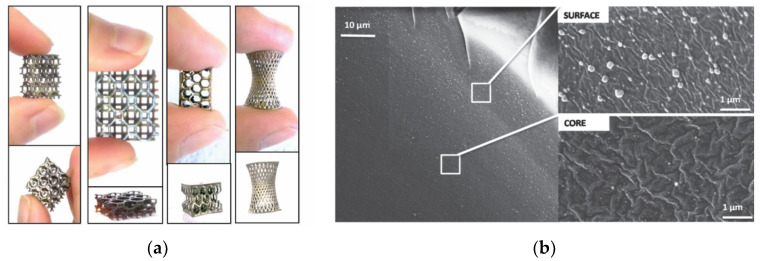
(**a**) Different types of Ag NPs-polymer composite 3D printed structures and (**b**) cross-section Figure 10. phr AgNO_3_ where can be seen the difference in the distribution of Ag particles in the surface and in the core. Reproduced from [73] with permission from Wiley.

Fantino et al. studied the in situ generation of silver nanoparticles by 3D-printing and subsequent UV postcuring [73]. The authors employed AgNO_3_ as the source for the silver nanoparticles in a PEG-diacrylate-based formulation. A radical photoinitiator with absorption in the near-visible range was used for the 3D-printing process in DLP equipment, and another radical photoinitiator with absorption in the UV range was used for the UV postcuring. The reason for this was the need for completion of acrylate polymerization after 3D-printing and to further promote an efficient reduction of silver during the UV postcuring. The aspect of the printed objects changed from semitransparent to silver mirrorlike after UV postcuring. Formation of Ag nanoparticles was confirmed by UV-vis spectroscopy and field emission scanning electron microscopy (FESEM). Silver nanoparticles with uniform and fine distribution within the samples were obtained, but larger particles and agglomerates were observed at the surface (see Figure 14). A decrease in electrical resistivity with increasing silver content, up to a maximum value with 15 phr of silver salt, was reported. Later, Fantino et al. studied the effect of a thermal treatment of 3D-printed objects [74] instead of a UV postcuring to promote the reduction of the metal precursor and possible sintering of the silver nanoparticles. According to UV-vis spectroscopy results, thermal treatment at higher temperatures promoted the formation of larger nanoparticles with closer packing. However, the increase in conductivity was far lower than with the previous UV postcuring treatment [73]. Thermal treatment under oxidative atmosphere at high temperature led even to degradation/oxidation, therefore worsening the conductivity of the materials. Particle sintering was not observed either.

**Figure 15:**
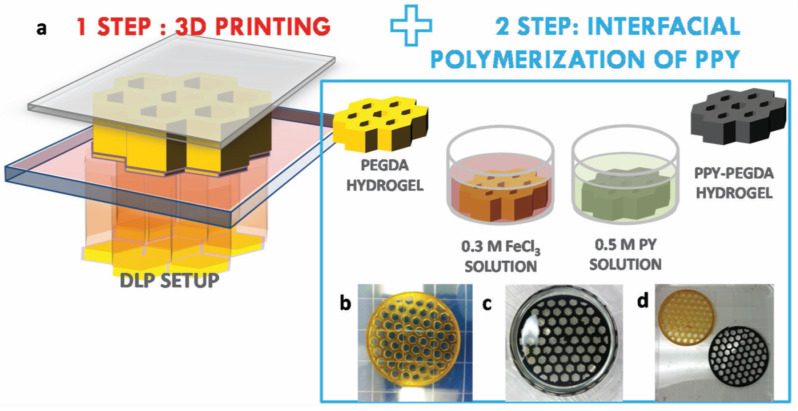
(**a**) Sketch of the production of electrically conductive hydrogels. (**b**) 3D printed PEGDA structure (**c**) PEGDA structure in PY/CYH solution. (**d**) 3D PEGDA and 3D PEGDA/PPY structures. Reproduced from [75] with permission from Wiley.

Electrically conductive hydrogels were also produced by Fantino et al. [75] following a two-step procedure consisting of (see Figure 15) (1) 3D-printing radical polymerization of PEGDA—water using radical photoinitiator and methyl orange dye followed by (2) interfacial polymerization (pyrrole oxidation). After the 3D-printing process, 3D-printed parts were soaked in ferric chloride solution followed by soaking in pyrrole (PY) solution in cyclohexane, producing pyrrole oxidation and polymerization. Interpenetration of the two polymers was reported. Loading of PY depended on initial water content of the printed hydrogel, allowing more room for diffusion and incorporation. The incorporation of PY effectively decreased the resistivity of the materials by one order of magnitude or more in comparison, and it was much lower than that of PEGDA photocured without water. However, the presence of trapped water in the samples already produced a significant decrease in resistivity, in the absence of PY. The presence of PY also increased the modulus of the produced materials.

**Figure 16:**
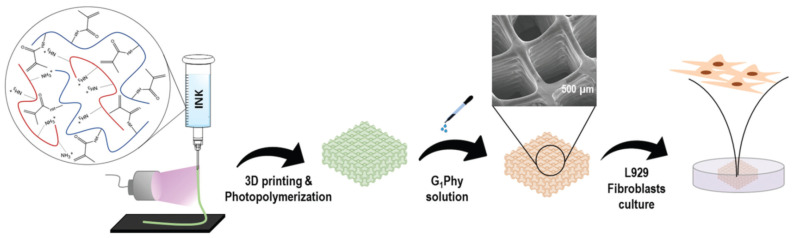
Schematic representation of the printing process applied for obtaining chitosan-methacrylated gelatin 3D polymeric scaffolds. Reproduced from [77]. Published by The Royal Society of Chemistry.

Multistage processing based on 3D-printing finds also application in the processing of biocompatible scaffolds making use of crosslinkable hydrogels. Pan et al. [76] produced cell-laden alginate microparticles and incorporated them into a photo-crosslinkable methacrylated hyaluronic acid hydrogel. A scaffold was produced by pneumatic extrusion of this hydrogel in a gelatin slurry support bath, followed by UV light exposure in order to produce the crosslinking of the hydrogel and removal of the gelatin support. Viability of the embedded cells and further proliferation after 3D-printing and UV-exposure were demonstrated. In contrast, Mora-Boza et al. [77] employed a different strategy for the production of multilayered scaffolds. Water solutions of chitosan-methacrylated gelatin were processed in a pneumatic extrusion 3D-printer, with UV irradiation on the needle outlet to initiate methacrylate polymerization, followed by ionic crosslinking by immersion in glycerylphytate solution or sodium tripolyphosphate (TPP) solution (Figure 16). The scaffolds crosslinked with glycerylphytate showed good cell viability and no cytotoxicity, in comparison with TPP.

**Figure 17:**
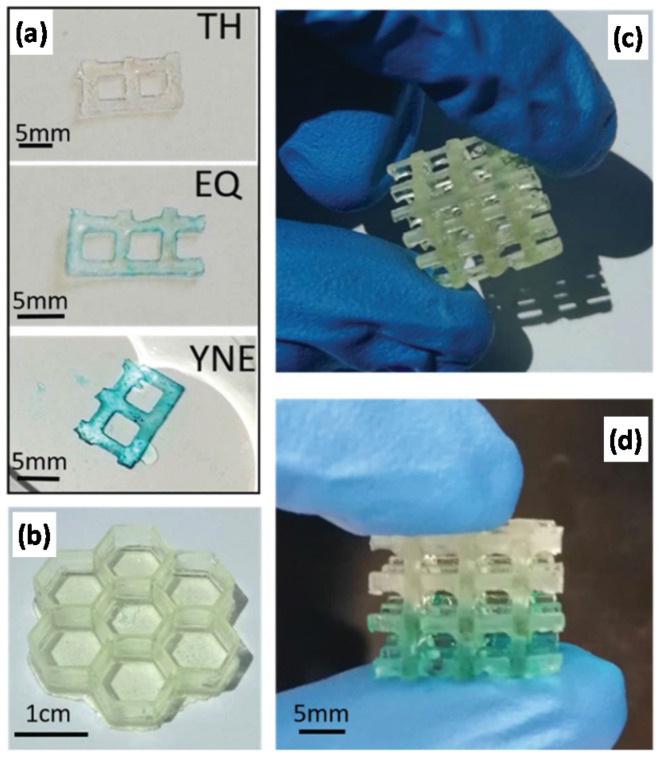
Illustrations of the 3D printed objects produced in [78]. (**a**) Result of the post functionalization step with the squaraine dye for the three thiol-yne formulations. (**b**) Structure built with the stoichiometric formulation (EQ), (**c**) hybrid structure with the two off stoichiometric formulations (YNE at the bottom and TH at the top), and (**d**) result of the post functionalization process on the same structure. Reproduced from [78] with the permission from The Royal Society of Chemistry.

Roppolo et al. [78] exploited the possibility of post-functionalization of –yne bonds to create 3D-printed objects with graded properties. The authors used thiol-yne formulations with different thiol-yne ratios that were printed in a DLP printer (Figure 17). The authors changed the composition of the resin formulation during the 3D-printing process, creating gradient properties. Gradient functionalization of unreacted –yne bonds was demonstrated with the help of an azide-terminated squaraine fluorescent dye in the presence of Cu.

**Figure 18:**
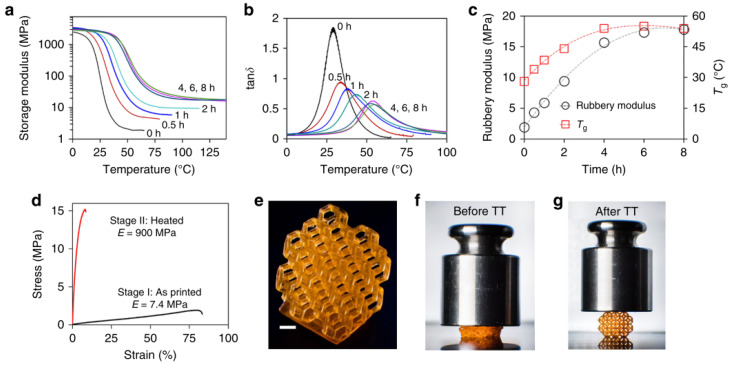
Demonstration of changes in stiffness of 3D printed thermosets produced in [79], caused by transesterification reactions leading to network structure rearrangement. (**a**–**c**) Dynamic mechanical analysis (DMA) showing the effect of the thermal treatment on the mechanical properties of samples. (**d**) Uniaxial tensile tests of printed samples before and after the 4 h thermal treatment at 180 °C. Demonstration of stiffness change of a printed Kelvin foam (**e**), before (**f**), and after (**g**) thermal treatment (TT) at 160 °C for 12 h. Adapted from [79], published under CC BY 4.0 license.

Another interesting concept is the application of a second treatment to produce a rearrangement of the material structure instead of a second polymerization. Zhang et al. [79] described a strategy for thermal post-processing of 3D-printed parts based on the bond exchange transesterification reactions taking place using a suitable catalyst at elevated temperature. These reactions introduced further crosslinking in the network during post-treatment, therefore enhancing mechanical properties, up to final equilibrium conditions (see Figure 18). In addition, the materials exhibited welding, healing and recycling capabilities not present in other 3D-printed materials. The structure-properties relationships associated with the structural rearrangement taking place during transesterification were recently modeled by Luo et al. [80]. This approach contrasts with that employed by Shi et al. [81], who developed inks for direct ink writing (DIW) based on DGEBA and fatty-acid precured vitrimer precursors. The printed objects were postcured at different temperatures to achieve materials with the desired ultimate properties. The materials could be fully recycled and reutilized in subsequent printing processes.

It is acknowledged that some of these approaches may fall beyond the dual-curing concept, and in consequence they would deserve a more detailed analysis within their specific application. However, they serve to illustrate different ways by which additional functionalities can be incorporated to 3D-printed materials by means of diverse post-polymerization processes, making it possible to explore novel application scenarios for 3D-printing processing.

## 6. Conclusions

Several dual-curing strategies employed in the processing of thermosets for 3D-printing applications have been reviewed: (1) reactivation of the radical homopolymerization of acrylates/methacrylates by means of a thermal initiator, (2) simultaneous activation of radical and cationic polymerization in the 3D-printing stage, (3) sequential thermal activation of the second polymerization after 3D-printing and (4) other post-functionalization processes. It has been shown that such strategies can serve to overcome some of the drawbacks of 3D-printing and the resulting materials, and in addition open new and exciting possibilities from the material design and application points of view, especially in the case of sequential dual-curing systems or sequential processing of 3D-printed structures.

Significant and exciting developments have been made so far, but taking into consideration the limited amount of publications, this research area is still largely unexplored. Further development and expansion of 3D-printing technologies will surely increase demand for materials with more demanding and tunable properties, and dual-curing systems can play a relevant role. Future research directions include but are not limited to: (1) the study of feasible combinations of reactive polymerization systems in 3D-printing scenarios, especially taking into consideration practical issues of production environments, (2) an in-depth analysis of structure-properties relationships of the intermediate and final materials to define sound design criteria, (3) optimization of 3D-printing and post-processing stages oriented to maximizing productivity without compromising the quality of the printed objects, (4) exploring the benefits and application potential derived from the existence of an intermediate material with controlled and stable properties in sequential dual-curing systems, and (5) studying post-polymerization strategies to enhance the functionality of printed objects, therefore expanding their scope of application.

## Data Availability

No new data were created or analyzed in this study. Data sharing is not applicable to this article.
